# Quantifying the behavioural consequences of shark ecotourism

**DOI:** 10.1038/s41598-023-39560-1

**Published:** 2023-09-07

**Authors:** Joel H. Gayford, William D. Pearse, Rafael De La Parra Venegas, Darren A. Whitehead

**Affiliations:** 1 Department of Life Sciences, Silwood Park Campus, Imperial College London, London, UK; 2 Shark Measurements, London, UK; 3 Ch’ooj Ajauil A.C., Cancún, Q. Roo México; 4 Investigación Tiburones México, La Paz, B.C.S. México

**Keywords:** Ecology, Zoology

## Abstract

Shark populations globally are facing catastrophic declines. Ecotourism has been posited as a potential solution to many of the issues facing shark conservation, yet increasingly studies suggest that such activity may negatively influence aspects of shark ecology and so further pressure declining populations. Here we combine UAV videography with deep learning algorithms, multivariate statistics and hidden Markov models (HMM) to quantitatively investigate the behavioural consequences of ecotourism in the whale shark (*Rhincodon typus*). We find that ecotourism increases the probability of sharks being in a disturbed behavioural state, likely increasing energetic expenditure and potentially leading to downstream ecological effects. These results are only recovered when fitting models that account for individual variation in behavioural responses and past behavioural history. Our results demonstrate that behavioural responses to ecotourism are context dependent, as the initial behavioural state is important in determining responses to human activity. We argue that models incorporating individuality and context-dependence should, wherever possible, be incorporated into future studies investigating the ecological impacts of shark ecotourism, which are only likely to increase in importance given the expansion of the industry and the dire conservation status of many shark species.

## Introduction

Sharks belong to the clade *Chondrichthyes*, an ancient radiation of crown group gnathostomes to persist in modern ecosystems^1^. Whilst sharks are of intrinsic interest due to their persistence through evolutionary time, they are also critically important components of marine ecosystems and are thought to be among the most functionally diverse vertebrate clades^2^. Amongst the ecological functions performed by sharks are the distribution of predation pressure through space and time^3^ and (in the case of migratory species) facilitation of energy transfer between ecosystems^2,4^. There is mounting evidence that declines in shark populations can result in phenomena such as mesopredator release and trophic cascades^5^. Despite their ecological importance, sharks are amongst the most threatened of all vertebrates, with recent IUCN (International Union for the Conservation of Nature) estimates suggesting that more than one third of all shark and ray taxa are facing extinction^6^. Overfishing is undoubtedly the major driver of this crisis, to which sharks are particularly vulnerable due to life history traits including relatively slow maturation and low fecundity^7^. This is not the only driver of decline, with anthropogenic climate change and habitat destruction also thought to be relevant in some populations^8,9^. The scale of the threats posed by overfishing and a general lack of public awareness have until recently provided major barriers to the implementation of conservation action^6,10,11^. Gradually these barriers are being lifted, with public perception increasingly favouring the protection of sharks^11^.

Shark ecotourism, in which members of the public pay to experience interactions with wild sharks, is credited with portraying sharks in a more positive light amongst members of the public^12,13^. Shark ecotourism is of ever-increasing economic importance in a number of countries, thought to be valued globally at over 300,000,000 USD per year and responsible for the creation of thousands of jobs^14,15^. Regardless of these clear socioeconomic benefits, ecotourism also has potential ecological impacts^16–19^, the true nature of which remain poorly understood. There is some evidence that ecotourism activities involving provisioning can influence both relative abundance and species composition^20^, and even directly trigger mesopredator release—increasing the abundance of other species at lower trophic levels^21^. Whilst trophic cascades are typically considered from the perspective of depredation, this is not strictly a requirement^22^, and thus it is possible that the feeding of sharks at ecotourism sites could result in functionally similar shifts in community ecology. Not all studies have recovered evidence for ecological impacts of ecotourism^23,24^, and even where present these potential effects are likely to be highly context dependent^16^. Even where provisioning is absent, disturbance and boat-related injuries remain substantial threats^16,25^. For these reasons, further studies are urgently warranted to establish the extent to which this expanding industry may have unforeseen ecological consequences on the populations it is aiming to conserve.

Whilst there are multiple potential routes by which shark ecotourism could influence their ecology, potentially the most important is through behavioural responses. Behaviour is the suite of traits by which inter-specific interactions are directly mediated^26^ and is thus a highly significant factor influencing downstream ecological consequences of anthropogenic interference. Even if such effects were limited to the focal taxon of ecotourism activities alone, disturbance and alterations to the landscape of fear have many potential consequences for bioenergetics, which in turn can have disastrous consequences for migration, reproduction, and other life-history traits^27,28^. Emerging studies are increasingly suggesting that behavioural plasticity may play a key role in dictating population-level resilience in the face of environmental change^29,30^. Several studies have attempted to address the effects of ecotourism on shark behaviour, reporting evidence of ecotourism influencing foraging activity, long-distance migratory behaviour and avoidance/disturbance responses^16–34^. Despite this, different studies present conflicting results, the interpretation of which is complicated by a lack of standardisation in the behavioural assays considered between studies. The temporal resolution of such studies also provides several limitations: some studies rely on telemetry data which, and although these are of unquestionable importance^35^, they do not typically consider the effects of ecotourism at the scale of individual behavioural sequences and interactions. Those that do typically consider behaviour to fall into one of a small number of qualitatively defined categories^34^. Using discrete, qualitative categories as opposed to rigorous quantitative definitions is not ideal as they are unlikely to be an accurate representation of the full repertoire of behavioural observed in most taxa. Studies utilising a more quantitative, biologically reasoned and quantitative approach and considering behaviour at finer temporal resolutions are essential if we are to fully understand how ecotourism activities may influence the behaviour (and subsequently the ecology and evolution) of sharks.

In this study we combine Unoccupied Aerial Vehicle (UAV) videography with deep learning algorithms, multivariate statistics and Hidden Markov Models (HMM) to investigate the ecological consequences of interactions between sharks and humans for shark behaviour, using whale sharks (*Rhincodon typus*) as a case study. This approach considers behavioural consequences of ecotourism for sharks at a finer temporal resolution than any previous study and uses explicit and rigorous quantitative definitions of behaviour. This increases potential for direct comparison between studies, aiding in ease of interpretation and considering the full range of movements observed during individual behavioural sequences. We comment on the implications of these results for ecotourism practices, for the ecology of the *R. typus* and the wider community.

## Methodology

The goal of this study was to amass aerial footage of *R. typus* both in isolation and in the presence of humans, quantify shark movement within the footage using neural networks, and establish the extent to which human activity influences the behaviour of *R. typus*.

### Ethics statement

Data collection and analysis procedures in this study complied with national animal welfare laws and ARRIVE guidelines and regulations; all data collection procedures were authorized by Mexican wildlife authorities under the permit SPARN/DGVS/04909/22 provided by the Comisión Nacional de Acuacultura y Pesca (CONAPESCA). This permit, issued by CONAPESCA is necessary and sufficient for all procedures conducted during this study (including the involvement of human and animal participants), and negates the requirement for IRB ethics approval, which is not required by Mexican law for such studies in Mexican territory.

### Data collection

**Figure 1:**
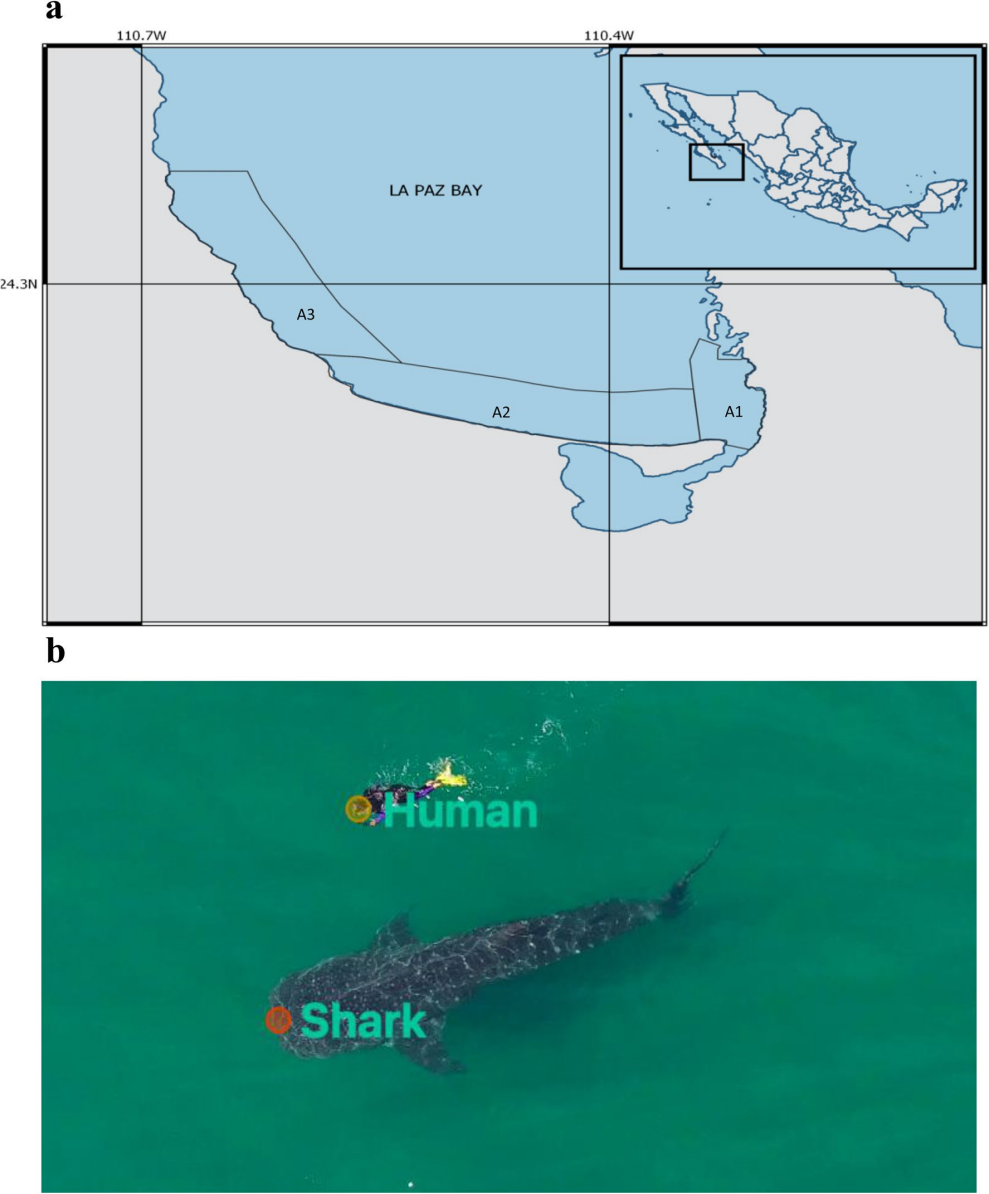
Images showing (**a**) the Bay of La Paz in Baja California Sur, Mexico where data collection took place; polygons A1, A2, and A3 represent the whale shark refuge area in which certain restrictions regarding boat traffic apply. (**b**) The labelling of humans and sharks for SLEAP analyses, with human swimmer mimicking ecotourist behaviour, swimming parallel to the shark maintaining a distance of at least two metres^37^.

Aerial videos of whale sharks and their interactions with ecotourism activities were obtained using a DJI Phantom 3SE UAV (flown at a constant altitude of 15 m), between the 30th of November 2022 and 6th of February 2023 (during the ecotourism season) in the whale shark refuge area in the Bay of La Paz, Mexico (Fig. 1a). This large, shallow bay hosts seasonal aggregations for juvenile whale sharks, which have become the focus of local ecotourism activities^34,36^. After filming sharks in isolation, a swimmer entered the water and mimicked typical ecotourist behaviour, swimming parallel to the shark with a minimum distance of two metres between them and the shark at all times. The position of the swimmer relative to the shark was variable, and depended both on the behaviour of the animal and the orientation of the vessel. One swimmer was used so as to increase the simplicity and intelligibility of our results, as otherwise additional potential confounding factors including size and behaviour of swimmers would need to be taken into account. Aerial videos were gathered both of sharks in isolation and interacting with swimmers. In total, 39 videos were obtained (20 with swimmers and 19 without), with video clips (following trimming) ranging from 167 to 1121 frames (5.6–37.4 s) in duration. All data was collected early in the day prior to the commencement of ecotourism activities. As aerial photographs are not typically used to assign IDs to whale sharks, we were unable to verify that each video corresponds to a unique individual.

### Video analyses

Aerial footage was analysed using the Python-based^38^ deep learning system SLEAP^39^, outputting coordinate data indicating the position of sharks and humans within the field of view of the drone. Sharks were modelled as a single point, corresponding to the anterior-most point on the midline of the dorsal body surface (Fig. 1b). Humans, where present, were labelled as a single point corresponding to the head (Fig. 1b). The use of a single point to model organisms minimised the potential impact of image distortion, which was deemed negligible following preliminary tests and extensive footage visualisation. Initially, 20 frames were labelled at random intervals within each video. SLEAP offers two neural network training modes: top-down training first identifies animals and then separately estimates the pose of each, whereas bottom-up training first identifies all of the body parts in a frame and separately assigns parts to each animal^39^. We chose a top-down mode following preliminary tests for training efficacy. The neural network was trained to predict the position of humans and/or sharks in 20 additional frames and ceased after five rounds of training, after which a manual review of the predicted frames was conducted. Following any necessary corrections (to the positions of misidentified sharks/humans estimated during the learning phase), the trained neural network was used to generate track data for all individuals across the entire video. To distinguish between humans and sharks, a centroid cost function was used, assigning identity on the basis of the distance travelled between frames. A Hungarian matching algorithm, which matches individual identity between frames by maximising total similarity^39^, was utilised to compile the final track data for each individual in each video.

### Parameter estimation

To obtain behavioural parameters for statistical analysis, SLEAP output was converted into trajectory data. Each frame transition in each video is modelled by a step length (indicating the distance between an individual’s location in two consecutive frames) and a turning angle (indicating change in directionality between two consecutive frames) using the *trajr* package in the *R* statistical environment^40,41^. As larger sharks are likely to swim faster than smaller sharks, a simple body size correction was applied to each trajectory in accordance with the literature^42,43^. Trajectories were visualised and smoothed accordingly (using a Savitzky-Golay filter of length 21 and polynomial order 3) in *trajr* prior to parameter estimation to remove noise associated with head yaw (lateral movements of the head), which could influence the distribution of parameters related to directionality. Smoothing parameters were chosen in line with previous studies and following visualisation of the data^41^. The following parameters were calculated for each video: minimum, maximum, mean and standard deviation values for speed, acceleration and turning angle; mean directional autocorrelation^44^, ‘eMaxA’^45^, ‘eMaxB’^45^ and ‘Sinuosity2’^46^. For the calculation of all trajectory-based parameters, a correlated random walk model of animal movement was applied under the assumptions of Brownian motion and that direction of movement in consecutive frames is correlated^47^.

### Statistical analyses

To test for statistical differences in the overall behavioural repertoire of individuals in the presence and absence of humans, an ‘ethospace’ visualisation was generated through principal component analysis (PCA). This approach is typically used to visualise ecological and morphological disparity^48^, but also provides a valuable tool by which multiple components of ‘behaviour’ can be compressed into a single ordination^49^. PCA analysis incorporating all parameters (*z*-transformed to account for scale differences between parameters) was performed using the packages *factoextra*^50^ and *ggplot2*^48,51^ in the *R* statistical environment^40^. Linear models were fitted for each of the parameters incorporated in the PCA in the *R* statistical environment^40^ and visualised using the package *ggplot2*^51^ using the presence or absence of humans as a binary predictor variable.

### Hidden Markov models

Whilst parameters such as mean speed, turning angle and acceleration are potentially biologically informative, they do not take into account the full range of behavioural plasticity exhibited by individuals. To increase the proportion of behavioural plasticity captured by the analyses whilst maintaining a biologically-reasoned approach that takes into account the ecology of the species in question, we applied discrete-time Hidden Markov models (HMM) to the non-smoothed trajectory data using the package *moveHMM*^40,52^ in the *R* statistical environment^40^. HMMs consist of a set of observations (in this case a series of step lengths and turning angles for each frame interval of each video), the distribution of which depends on the distribution of the hidden state (a proxy for individual behaviour, which takes one of a predefined set of discrete values at any given frame interval) which ‘evolves’ over time as a Markov process, described by a matrix of transition probabilities between each of the possible state values^53^. We applied an HMM to the data incorporating the presence/absence of humans as a binary covariate, tested against a null model without covariates. Step length and turning angle were modelled by Gamma and Von Mises distributions respectively^52^, defined by the parameters mean step length ($\mu$), step length standard deviation ($\sigma$), mean turning angle ($\theta$) and angle concentration factor ($\kappa$)—a measure of how centred turning angles are around zero^52^. Plausible parameter ranges were identified by visualising the actual step length and turning angle distributions, with these ranges being incorporated into a numerical likelihood optimization routine^54^ to identify parameter values corresponding to the akaike information criterion (AIC) global optimum. AIC balances model fit with model complexity^55^ and thus this global optimum should represent the model that explains the greatest proportion of variance in the data whilst maximising the simplicity and interpretability of the model. As some behavioural sequences featured frames in which no movement occurred, zero mass parameters ($\zeta )$ were incorporated into each model, the value of which corresponded to the proportion of steps of length zero in the dataset. Seventy five sets of parameter values (randomly selected within the boundaries of plausible values defined previously) were considered for each HMM (including one-state, two-state and three-state models), with final models selected on the basis of AIC and log likelihood scores^55^. Multinomial logistic regression was then performed on the model of best fit to quantify the effects of shark-human interactions on each of the transition probabilities between the three behavioural states. All HMM analyses assumed a correlated random walk model of animal movement in accordance with the literature^47,52^.

## Results

### Ethospace occupation of *R. typus*

**Figure 2:**
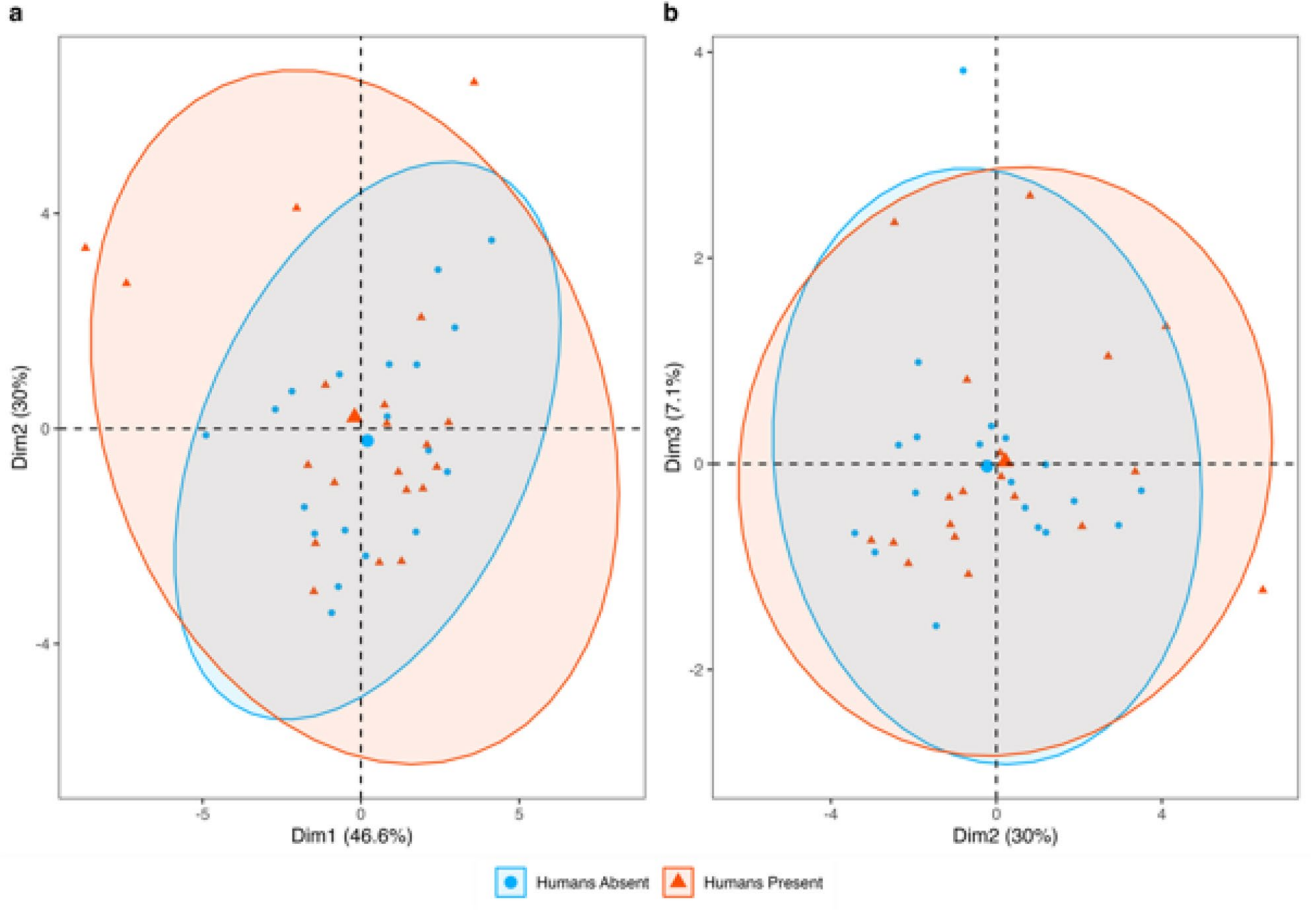
There is no difference in mean behaviour between individuals interacting with humans and those not interacting with humans. The total plot space represents the range of possible behaviours an individual could exhibit, with each data point representing a separate individual. Enlarged points represent mean position in ethospace of each group (blue points indicate the absence of humans whereas orange points indicate the presence of humans). As the mean value for each group overlaps with the 95% confidence ellipse of the other group, there is no statistically significant difference between the mean values.

PCA incorporating 16 behavioural variables did not recover any evidence of significant behavioural differences in the presence or absence of humans (Fig. 2). Whilst individuals in the presence of humans qualitatively appear to occupy a greater ethospace range, there is no statistically significant difference (as evidenced by overlapping confidence ellipses, *p* ≥ 0.05) between the mean behaviour of the groups (Fig. 2). The first three principal components cumulatively explained 83.7% of observed behavioural variance (46.6, 30.0 and 7.1% respectively), with the parameters standard deviation of speed (Dim 1, 9.9%), mean speed (Dim 2, 16.9%) and minimum angle (Dim 3, 74.9%) explaining the greatest proportion of variance in each principal component respectively.

### Linear models of behavioural variables

Linear regression of 16 behavioural variables against a binary variable representing the presence or absence of humans failed to recover evidence of statistically significant relationships between the presence of humans and the behaviour of *R. typus* individuals (Table 1).

**Table 1:** Regression output for each parameter against a binary variable representing human presence/absence, where a value of 0 represents human absence and a value of 1 represents human presence.

Parameter	Coefficient	Intercept	T value	*p* value	Standard error	Residual error	${R}^{2}$(%)	Adjusted ${R}^{2}\left(\text{\%}\right)$	df	F value
Mean speed	8.29E–03	1.04E–01	4.66E–01	6.44E–01	1.7799E–02	5.5560E–02	0.58	− 2.1	1,37	2.17E–01
Min speed	2.45E–03	1.99E–02	2.60E–01	7.96E–01	9.4160E–03	2.9390E–02	0.18	− 2.52	1,37	6.76E–02
Max speed	5.11E–02	2.23E–01	1.06E + 00	2.95E–01	4.8150E–02	1.5030E–01	2.96	0.33	1,37	1.13E + 00
Speed SD	7.51E–03	3.16E–02	9.34E–01	3.56E–01	8.0380E–03	2.5090E–02	2.3	− 0.34	1,37	8.73E–01
Mean acceleration	2.00E–03	1.30E–02	6.94E–01	4.92E–01	2.8890E–03	9.0170E–03	1.28	− 1.39	1,37	4.81E–01
Min acceleration	− 2.00E–06	8.10E–05	− 4.30E–02	9.66E–01	4.5000E–05	1.3900E–04	0.01	− 2.7	1,37	1.88E–03
Max acceleration	2.09E–02	7.29E–02	8.23E–01	4.16E–01	2.5330E–02	7.9080E–02	1.8	− 0.86	1,37	6.78E–01
Acceleration SD	2.28E–03	1.10E–02	8.16E–01	4.20E–01	2.7910E–03	8.7110E–03	1.77	− 0.89	1,37	6.65E–01
Mean angle	5.24E–03	1.64E–01	3.01E–01	7.65E–01	1.7416E–02	5.4360E–02	0.24	− 2.45	1,37	9.04E–02
Min angle	− 5.70E–05	3.84E–04	− 4.11E–01	6.83E–01	1.3800E–04	4.3100E–04	0.46	− 2.24	1,37	1.69E–01
Max angle	− 1.49E–01	1.86E + 00	− 4.64E–01	6.45E–01	3.2090E–01	1.0020E + 00	0.58	− 2.11	1,37	2.16E–01
Angle SD	− 1.73E–02	2.16E–01	− 5.00E–01	6.20E–01	3.4490E–02	1.0770E–01	0.67	− 2.01	1,37	2.50E–01
Directional autocorrelation	− 1.51E–02	8.21E–01	− 3.48E–01	7.30E–01	4.3210E–02	1.3490E–01	0.33	2.37	1,37	1.21E–01
eMaxA	− 4.22E + 00	4.90E + 01	− 3.68E–01	7.15E–01	1.1478E + 01	3.5830E + 01	0.37	− 2.33	1,37	1.35E–01
eMaxB	− 1.86E–01	5.61E + 00	− 8.60E–02	9.32E–01	2.1535E + 00	6.7220E + 00	0.02	− 2.68	1,37	7.45E–03
Sinuosity2	− 3.15E–02	8.37E–01	− 2.90E–01	7.73E–01	1.0853E–01	3.3880E–01	0.23	− 2.47	1,37	8.42E–02

### HMM state allocation and model fit

HMMs including three discrete behavioural states received more support than models including either one or two behavioural states ($\Delta$AIC ≥ 14,202, $\Delta$LL (maximum log-likelihood) ≥ 7115. The model of best fit incorporating human presence/absence as a covariate received significantly more support (in terms of AIC and log-likelihood values) than null models not accounting for human activity (Table 2). On the basis of step length and turning angle ranges we define the three states incorporated into our HMM as follows: State 1 covers relatively low step lengths and a high angle concentration factor (Table 2), reflecting highly directed movement at relatively low velocity^54^, such as may be observed when transiting between areas of high prey density. State 2 covers relatively large step lengths and a relatively low angle concentration factor (Table 2), reflecting high velocity and highly angular movement, such as might be expected in predator escape responses and avoidance/disturbance behaviour. State 3 covers intermediate step lengths and an intermediate angle concentration factor (Table 2), encompassing the ranges of velocity and angularity observed during both resting and surface feeding behaviours. Henceforth these states will simply be referred to as State 1, 2 and 3 to avoid controversy regarding the use of subjective terminology to describe behaviour. The biological interpretations of these states must be treated as hypotheses based on the quantitative definitions of each state, which are framed with respect to the velocity and angularity of trajectories. Transitions between states will be referred to as State $x\to y$, where $x$ is the initial state and $y$ is the final state.

**Table 2:** HMM model of best fit including presence/absence as a covariate. Model fit determined on the basis of the difference between the Akaike Information Criterion (AIC) and log-likelihood (LL) values of covariate models and null models ($\Delta$AIC and $\Delta$LL). State parameters refer to mean step length, step length standard deviation, mean turning angle, angle concentration factor and zero mass parameter as defined in the methodology.

Model covariate	AIC	$\Delta$AIC	LL	$\Delta$LL	State 1	State 2	State 3
Presence/absence	19,539	140	− 9741	76.2	$\mu$: 2.21E–02 $\sigma$: 1.37E–02 $\theta$: 1.53E–02 $\kappa$: 1.10E + 00 $\zeta$: 9.73E–04	$\mu$: 3.76E–01 $\sigma$: 3.64E–01 $\theta$: − 1.15E + 00 $\kappa$: 4.22E–02 $\zeta$: 7.26E–03	$\mu$: 2.74E–01 $\sigma$: 6.93E–02 $\theta$: − 2.15E–02 $\kappa$: 6.09E–01 $\zeta$: 4.11E–11

### HMM state occupancy and multinomial logistic regression

**Figure 3:**
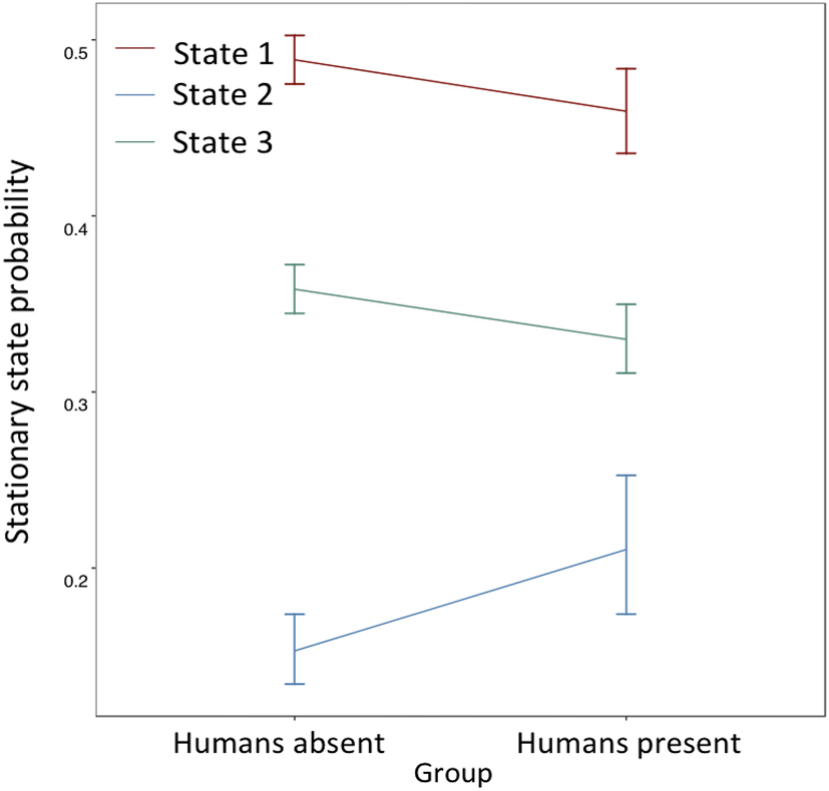
Human presence significantly influences the long-term probability (stationary state probability) of individuals occupying a behavioural state characterised by high speed and high angularity. Red represents state 1, blue represents state 2 and green represents state 3. Error bars represent 95% confidence intervals for the respective datapoints.

**Figure 4:**
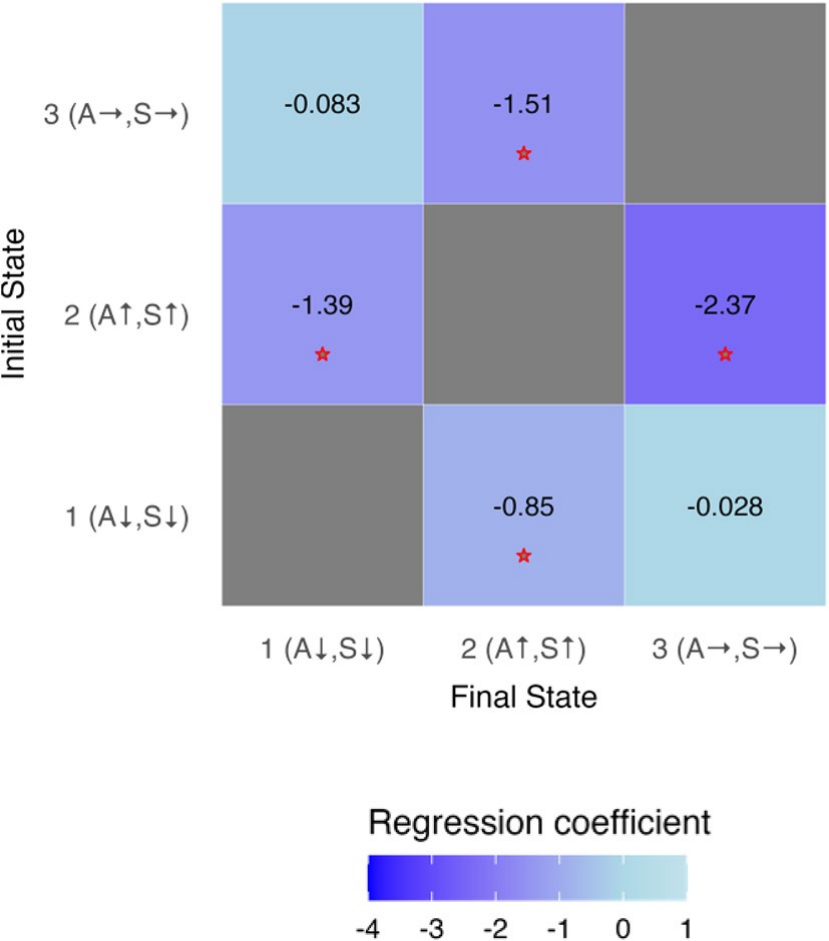
Multinomial logistic regression coefficients demonstrate that human presence/absence (regression treats this as a binary variable with human absence holding a value of zero and human presence a value of one) significantly alters the probability of transitioning between some (but not all) behavioural states. A and S refer to the relative angularity and speed of each state, where ↑, ↓, and → represent relatively high, low and intermediate values. Red stars denote relationships found to be significant (*p* ≤ 0.05).

The human presence/absence model suggests that regardless of whether humans are present, the long-term probability of an individual being in State 1 (typified by highly directional movement) is greater than that of an individual being in State 3 (typified by intermediate speed and directionality), which is in turn greater than the probability of an individual being in State 2 (typified by rapid, erratic movement consistent with escape behaviour) (Fig. 3). However, the presence of humans results in an increase in the long-term probability of an individual being in State 2 (Fig. 3). Whilst human presence appears to result in a decrease in the long-term probability of an individual being in either State 1 or State 3, these relationships were not found to be significant. Multinomial logistic regression applied to this model suggests that human presence has a significant influence on all transition probabilities except State $3\to 1$ and State $1\to 3$ (Fig. 4).

## Discussion

Our results demonstrate that the presence of humans and their proximity to *R. typus* individuals has important behavioural consequences for these sharks, and that these consequences are only detected by models that account for hidden behavioural states and individual variation in behaviour (Figs. 3 and 4). Of particular significance with regards to ecology and conservation, ecotourism activity increases the probability of individuals being in a disturbed behavioural state typified by relatively angular and rapid movement (Fig. 3). Moreover, ecotourism activity significantly influences the probability of transitioning between states (Fig. 4), specifically reducing the probability of transitioning from a disturbed to an undisturbed state (Fig. 4: State $2\to 1$, State $2\to 3$). Whilst it may appear counter-intuitive that the probability of leaving State 2 is also increased in the presence of humans (Fig. 4), this is consistent with reductions in velocity and angularity following completion of a disturbance response. It is categorically not evidence of ‘relaxation’ or the absence of disturbance responses in the presence of humans, and is rather a ‘return to normality’ following successful evasion of a perceived threat. This is supported both by the other transition probabilities (Fig. 4), and qualitative observation of video footage. The primary behavioural consequence of shark ecotourism for *R. typus* individuals appears to be an increase in the proportion of time spent in a disturbed state typified by increased energetic expenditure^56^, relative to states encompassing less rapid and angular movements (Figs. 3 and 4).

Avoidance behaviours increase energetic expenditure as a result of the energy required to generate such behaviours^56^, however expenditure also increases indirectly as a result of displacement of individuals from areas of high foraging success^57,58^. Whilst reduction in the prevalence of foraging behaviour in whale sharks in the presence of humans has previously been reported^34^, we recovered evidence of increases in energetic expenditure as human presence significantly increased the long-term probability of individuals engaging in behaviours typically associated with avoidance/disturbance (Fig. 3). Such displacement would incur fitness costs in any species, however *R. typus* individuals aggregate in the Bay of La Paz specifically to forage^36,59^ and have been known to engage in vast oceanic migrations^60,61^. For these reasons reduced foraging success in the Bay of La Paz may be particularly impactful in terms of bioenergetic fitness consequences. Indeed a recent study of whale shark bioenergetics indicated that the presence of humans engaging in ecotourism activities results in a significant increase in metabolic rate in whale sharks^62^. Whilst no such study has been conducted in the Bay of La Paz, the behavioural observations reported are consistent with our results. Moreover, as whale sharks in La Paz are not provisioned (unlike those in the bioenergetic study mentioned) the metabolic consequences of ecoutourism may be even more significant^62^. In addition to the physiological consequences of reduced foraging success^63–65^, these behavioural changes could reduce reproductive success through modification of reproductive phenology^57^ and increase the risk of injuries associated with boat strikes^25,66^ given that even minor displacement would see individuals exit the protected area^34^. Inferring population-level consequences of these individual behavioural responses is not trivial^67^, however temporally persistent avoidance behaviours at the population level can result in area abandonment^68^, in turn triggering cascading ecological effects that influence entire communities^57^. Migratory sharks such as *R. typus* are known to act as a major biological nutrient flux between isolated ecosystems^2,4,69^, and thus area abandonment could have profound long-term consequences for nutrient cycling^69^. Agent-based models have been developed to predict population-level consequences of disturbance responses similar to those reported in this study^63,67^, however most of these studies consider marine mammals, and as of yet none have been applied to elasmobranchs. For this reason, the details of such consequences in *R. typus* and other elasmobranch populations remain poorly constrained and should be a focus of future work.

Whilst the relationship between bioenergetics and disturbance responses may appear straightforward, behavioural responses to disturbance (and their ecological consequences) are often highly nuanced and context-dependent^70,71^. An individual displaying an ‘undisturbed’ behavioural state does not necessarily imply that the stimulus in question is not adversely affecting this individual. Various physiological stress responses are known in a range of taxa^71–74^, many of which are thought to incur fitness costs even in the absence of obvious behavioural effects. Stress physiology has been studied in elasmobranchs^75^, but not in the context of ecotourism. Many studies have reported evidence of a relationship between individual behaviour (typically in the context of foraging behaviours or predator avoidance) and past history of energetic states and ecological interactions—often termed ‘experience’^68,76–79^. The utility of terms such as ‘personality’ and ‘experience’ to behavioural ecology is debatable^80,81^, however the initial behavioural state of individuals can often be of great importance in determining their response to a given stimulus. Our results support this concept as behavioural responses differed between *R. typus* individuals (Fig. 2) and the initial behavioural state of individuals was found to be important in determining the way in which they respond to human presence (Fig. 4). Behavioural differences in the presence and absence of humans were only detected when fitting models that account for individual variation in behaviour, and these models are supported despite their increased complexity (Table 2). Consequently, the behavioural, physiological and ecological consequences of a single disturbance event are not limited to the immediate time interval in which disturbance occurs but may persist for some as of yet undefined duration. This also raises the question of state-behaviour feedback, which has been reported in other taxa^82–84^. *R. typus* individuals aggregating at common ecotourism sites are likely to experience a number of interactions with humans in any given day, and if disturbance responses demonstrate synergism then the true ecological consequences of ecotourism in this taxon may be far greater than previously considered. Further studies will be required to elucidate the extent of the relationship between past behavioural/energetic context and contemporary *R. typus* behaviour, however we suggest that shark behaviour should always be assessed prior to ecotourism activity to minimise potential disturbance. Even if this advice is heeded, these results suggest that some negative ramifications of ecotourism may be unavoidable unless such activity ceases entirely.

Our results demonstrate the importance of utilising multiple statistical approaches in the analysis of behavioural data. On the basis of individual parameters such as mean acceleration or standard deviation of turning angle, one might suggest that ecotourism has a negligible impact on the fine-scale behaviour of *R. typus* (Table 1). This lack of responsiveness is not recovered when instead using Hidden Markov Models that account for individuality and past behavioural context (Fig. 4). Previous studies have produced superficially similar results^34^ but do not provide comparable temporal resolution, and do not utilise a fully quantitative approach. Moreover, both multinomial logistic regression and ethospace reconstruction reveal individuality and context dependence to *R. typus* behavioural responses (Figs. 2 and 4) which has not previously been reported. Whilst an HMM approach has been applied previously to shark spatial ecology^85–88^, this study is (to our knowledge) the first to use such a method in the context of high-resolution shark movement data. We suggest that this approach should form an important component of future studies, without which the nuanced and context dependent nature of behavioural responses to human activity may be neglected entirely. Particularly valuable additions to the literature would be studies considering relationships between behavioural responses to ecotourism and sex/ontogeny, factors we were unable to consider in the present study.

The importance of behavioural studies to ecology and conservation has long been understood^89^. By quantifying behaviour and applying multiple statistical approaches to these data, we have demonstrated that the influence of human activity on *R. typus* behaviour is significant, profound and context dependent. These behavioural consequences of ecotourism have potentially concerning implications for *R. typus* ecology. In light of these results, we suggest that the initial behavioural state of individuals must be assessed prior to in-water ecotourism activities, and that regulations regarding the minimum distance between human and shark should be revisited and reviewed in detail. In particular, we suggest that sharks engaging in rapid, angular movements should be avoided. Future studies investigating the relationship between ecotourism and elasmobranch behaviour should strive to use a biologically reasoned and rigorously quantitative approach wherever possible. Such studies will form an integral component of global efforts to conserve and protect declining elasmobranch populations, and as such ensuring reproducibility and ease of interpretation between studies should be of utmost importance.

## Data Availability

All data (the digitised tracks and estimated behavioural parameters) and code used in this project can be found in the following repository: https://figshare.com/s/bc287179a0b85c1c797c (to be made public following acceptance). Raw video footage can be provided upon reasonable request. They are over 9 GB in size and thus difficult to share online, however an exemplar video at low resolution has been deposited in the repository above.
